# Therapeutic Efficacy of Mirogabalin in managing Trigeminal Neuralgia

**DOI:** 10.4317/jced.61552

**Published:** 2024-05-01

**Authors:** Souichirou Tadokoro, Naoki Otani, Andrew Young, Kana Ozasa, Noboru Noma

**Affiliations:** 1 DDS, Department of Oral Medicine, Nihon University school of Dentistry Tokyo, Japan; 2 PhD, MD, Department of Neurological Surgery, Nihon University School of Medicine, Tokyo, Japan, 1-6 Kandasurugadai, Chiyoda-ku, Tokyo 101-8309, Japan; 3 MSD, DDS, Department of Diagnostic Sciences, Arthur Dugoni School of Dentistry, University of the Pacific, San Francisco, United States; 4 PhD student, Department of Oral Medicine, Nihon University school of Dentistry Tokyo, Japan

## Abstract

Trigeminal neuralgia presents significant challenges in management, often requiring alternative pharmacotherapy due to resistance or side effects to first-line medications like carbamazepine. This case series investigates the efficacy and safety of mirogabalin, a novel α2δ ligand, in six trigeminal neuralgia patients. Mirogabalin demonstrated varying degrees of pain reduction, with an average Numerical Rating Scale improvement rate of 43.1%. Side effects were generally mild, with drowsiness and dizziness being the most common. Despite limited efficacy in some cases, mirogabalin shows promise as a potential treatment option for trigeminal neuralgia, warranting further investigation.

** Key words:**Trigeminal neuralgia, mirogabalin, α2δ lig.

## Introduction

In 2020, the International Classification of Orofacial Pain (ICOP) refined the classification of classical trigeminal neuralgia, delineating it into two distinct categories: “classical trigeminal neuralgia, purely paroxysmal” and “classical trigeminal neuralgia with concomitant continuous pain” (1). The first-line pharmacotherapy for trigeminal neuralgia is carbamazepine; however, if resistance to or side effects from the medication occur, alternative drugs are necessary.

 Mirogabalin, a gabapentinoid medication approved in Japan, selectively targets the α2δ1 subunit of voltage-gated calcium channels, which is up-regulated in neuropathic pain (2). It exhibits higher affinity and slower dissociation from α2δ1 compared to pregabalin, resulting in potent and long-lasting pain relief. Mirogabalin is effective in conditions like postherpetic neuralgia and diabetic neuropathy, but its efficacy in trigeminal neuralgia requires further study (3,4). In this case study, we for the first time describe the clinical effectiveness of mirogabalin for trigeminal neuralgia.

## Case Report

The cases presented here are six patients with trigeminal neuralgia who visited the pain clinic at the Nihon University School of Dentistry Hospital, and were treated with mirogabalin. We recorded the gender, age, duration of illness, diagnosis based on ICOP, treatment efficacy, and side effects. Microgabalin was administered at doses of 5-30 mg/day. Pain severity was evaluated using the Numerical Rating Scale (NRS; 0 = no pain, 10 = worst pain imaginable) before beginning microgabalin, and after discontinuing microgabalin (usually 2-3 months after starting). The NRS improvement rate was expressed as a percentage using the formula: Improvement rate = 1 – (Final NRS / NRS at baseline).

-Case 1

A 42-year-old male presented with the chief complaint of paroxysmal pain in the right mandible.

**Figure 1:**
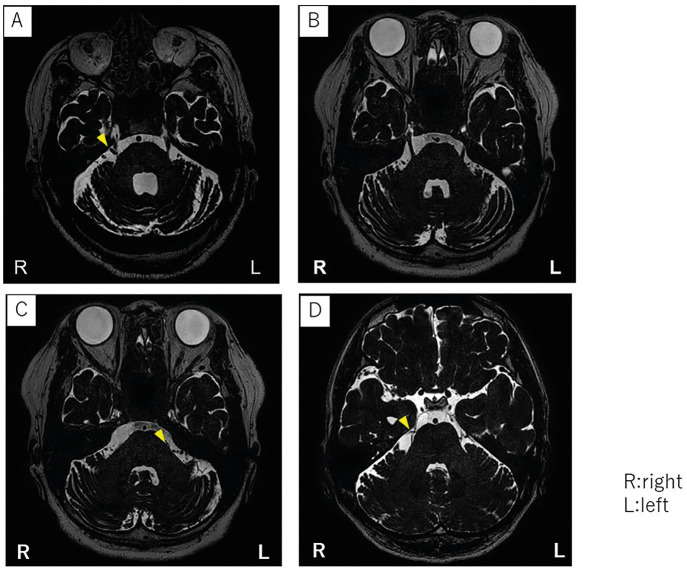
A-D) MRI scans showing compression of the trigeminal nerve by the superior cerebellar artery (SCA). R: right, L: left.

The initial onset was 9 months prior. One month before presenting at our clinic, he developed paroxysmal pain in the entire lower right jaw, lasting 1–2 min while eating or talking. Magnetic resonance imaging (MRI) revealed neurovascular compression at the root entry zone (Fig. 1). A clinical diagnosis of trigeminal neuralgia was made, and carbamazepine was started, but then discontinued due to liver dysfunction. The patient started oral mirogabalin 5 mg twice daily, and eventually increased it to 15 mg twice daily. The pain intensity on the NRS decreased from 8 to 3. With the patient’s consent, microvascular decompression surgery was performed, which eliminated the pain.

-Case 2

A 54-year-old female presented to our clinic with the chief complaints of electric shock-like pain on the right side of her face. She first noticed this pain approximately a year prior while applying makeup, experiencing it from the lower right jaw to the temple. She sought consultation with neurosurgery and was diagnosed with trigeminal neuralgia. Carbamazepine was prescribed initially, which provided some relief, but as the pain recurred, she was referred to her dentist and subsequently visited our clinic. Under our direction, she has been taking mirogabalin 5 mg twice daily, resulting in an improvement from a NRS score of 10 to 5. The mirogabalin dosage is currently being gradually increased to 15 mg, and the patient considers the progress to be satisfactory when she uses topical xylocaine concurrently.

-Case 3

A 74-year-old female presented to our clinic for severe pain in the right orbital region. Several years prior, the patient began experiencing electric shock-like pain lasting for one minute on the right side of her face during meals, conversations, and while applying makeup. She sought consultation with anesthesiology in southern Japan, where she was prescribed carbamazepine for trigeminal neuralgia, which initially provided relief. However, as the medication’s efficacy diminished over time, she was referred to our clinic when she relocated. She was prescribed mirogabalin 5 mg twice daily, which she took for a month with no improvement. We suggested increasing the dosage, but the patient requested radiofrequency thermocoagulation (RFT), which was performed on the infraorbital nerve, resulting in an elimination of the pain.

-Case 4

A 65-year-old female presented with paroxysmal pain on the left side of the mandible. She first noticed this pain five months prior to her visit to our clinic and underwent pulpectomy of the left mandibular premolar at a general dental clinic. Before seeking consultation at our clinic, she began experiencing intense pain during facial cleansing. MRI revealed neurovascular compression at the root entry zone (Fig. 1B,C). Under the diagnosis of trigeminal neuralgia, treatment was initiated with carbamazepine 100 mg, which initially improved the pain; however, it was discontinued due to renal dysfunction. The medication was then switched to mirogabalin, starting at 2.5 mg twice daily, resulting in no improvement of the NRS score of 10. Due to difficult pain control, we referred the patient to the Division of Neurosurgery.

-Case 5

A 73-year-old male visited our clinic complaining of stabbing pain in the right facial area. He first noticed intense stabbing pain from the right mandible to the face 10 months prior. The pain lasted for several minutes and was triggered by eating and talking. After undergoing an MRI and consulting with a neurosurgeon, no neuroanatomical abnormalities were observed. With the diagnosis of trigeminal neuralgia, carbamazepine treatment commenced at 100 mg, initially providing pain relief, but ceased due to renal dysfunction. Subsequently, the patient was transitioned to mirogabalin, with an initial dose of 5.0 mg twice daily. This led to a decrease in the NRS pain score from 10 to 1. At present, the pain is effectively managed with the same dosage.

-Case 6

A 71-year-old female presented with severe pain in the upper right maxilla. He first experienced episodic pain in the right maxillary second molar while eating two months prior, and underwent root canal treatment (RCT) in the Department of Endodontics. The episodic pain persisted, leading to referral to our clinic. Neurovascular compression at the root entry zone was identified through MRI (Fig. 1D). With the diagnosis of trigeminal neuralgia, carbamazepine was prescribed initially, which provided relief, but was discontinued due to side effects such as dizziness. Mirogabalin 2.5 mg twice daily was then prescribed, resulting in a decrease in the NRS score from 10 to 4. Currently, the pain is effectively controlled with the same dosage, ( Table 1).

## Discussion

Mirogabalin is a selective oral α2δ ligand belonging to the gabapentinoid class of neurological drugs, and was first approved in Japan in 2019 for the treatment of neuropathic pain (5). In a Phase 3 study involving patients with postherpetic neuralgia, mirogabalin was effective and well-tolerated (3). Another Phase 3 trial on patients with diabetic neuropathy showed a dose-dependent improvement in average daily pain scores after 14 weeks of treatment, with mild to moderate side effects (4).

Data on the efficacy of mirogabalin in the treatment of trigeminal neuralgia is not yet available, but some studies have been done with gabapentin and pregabalin (6,7). Considering the similarity in mechanism of action between gabapentin and pregabalin, and mirogabalin, similar effects in reducing neuropathic pain are expected. Cheshire *et al*. reported that among 92 trigeminal neuralgia patients taking gabapentin, 43 individuals experienced partial to complete relief in paroxysmal pain with a mean daily dose of 930 mg/day (range 100–2400 mg/day) divided into three doses per day (6). Obermann *et al*., for initial responses to pregabalin, observed an elimination of pain in 25% of patients, and a >50% reduction in pain in 49% of patients, at a mean dose of 269.8 mg/day (range 150–600 mg/day) (7).

In this study, cases 3 and 4 showed no effect with microgabalin; however, the average NRS value for all cases improved from 9.6 ± 0.8 before administration to 5.5 ± 3.7 at the final follow-up, demonstrating an improvement rate of 43.1 ± 36.4% on the NRS. Regarding side effects, previous studies reported the most frequent side effect was drowsiness, followed by vestibular dizziness, and no serious adverse effects (3). The side effects confirmed in this study were drowsiness and dizziness, with no serious adverse effects. From these results, mirogabalin can be investigated as a novel neuropathic pain medication due to its relatively low-dose analgesic effect and minimal side effects in trigeminal neuralgia.

## Figures and Tables

**Table 1:** Summary of cases presented in this series.

Case	Diagnosis	Age (years)	Sex	Duration of monitoring	Mirogabalin (total dosage/day)	Side effects for Mirogabalin	NRS at baseline	NRS at final	NRS improvement rate (%)	Previous medication or treatment
1	Classical trigeminal neuralgia, purely paroxysmal	42	Male	9 months	30 mg	None	8	3	62.5	Carbamazepine Pregabalin
2	Classical trigeminal neuralgia with concomitant continuous pain	54	Female	12 months	10 mg	None	10	5	50	Carbamazepine
3	Classical trigeminal neuralgia, purely paroxysmal	74	Female	36 months	10 mg	Dizziness	10	10	0	Carbamazepine
4	Classical trigeminal neuralgia with concomitant continuous pain	65	Female	5 months	5 mg (Renal dysfunction)	None	10	10	0	Carbamazepine
5	Classical trigeminal neuralgia, purely paroxysmal	73	Male	10 months	10 mg	Drowsiness	10	1	90	Carbamazepine Extraction Denture adjustment
6	Classical trigeminal neuralgia, purely paroxysmal	71	Female	2 months	5 mg (Renal dysfunction)	None	10	4	60	Carbamazepine Rootcanal treatment

## Data Availability

The datasets used and/or analyzed during the current study are available from the corresponding author.
